# Extra Supplement—The Nursing Mirror

**Published:** 1890-08-30

**Authors:** 


### "The Hospital" Nursing Mirror.

Being the Extra Nursing Supplement of "The Hospital" Newspaper.

All Contributions for this Supplement should be addressed to the Editor, The Hospital, 140, Strand, London, W.C., and should have the word "Nursing" plainly written in left-hand top corner of the envelope.

### En Passant.

SCARBOROUGH INSTITUTE.—This Institute and Home Hospital has completed the second year of its existence. During last year the nurses attended 200 cases, but owing to the smallness of the staff, which has been twice increased, a large number of calls for nurses had to be refused. A larger house has been taken during the course of the year.

WORK AT WATFORD.—A report has lately been issued of the six years' work of the district nurses of Watford. There are two nurses, Miss L. H. Scott, and an assistant, and last year over 4,000 visits were paid. Of the 174 cases nursed, 120 recovered, 31 died, and 23 are still on the books. The committee speak very highly of the nurses' work, which has this year been recognised by a liberal donation from the Hospital Demonstration Committee.

GOOD NEWS FOR CHERTSEY.—The Local Government Board has made a communication to the Chertsey Board of Guardians about the necessary care and room for the sick at the workhouse. Amongst other recommendations is the following :—"The nursing in the infirmary should be performed by four nurses. Paupers should be brought from the house to do scrubbing, etc., but should on no account remain assistants to the nurses. The head nurse in charge should be well qualified, have a separate room, suitable dress, and a salary of £35 a-year. The other nurses should have £25 a-year, dress, and suitable accommodation ; one of these nurses should be always on duty at night."

QUEEN VICTORIA NURSES.—Lady Rosebery, ever thoroughly interested in nursing work, has visited the new Home at Edinburgh, which is now occupied by the Jubilee nurses. There are rooms for twelve nurses, and the number can be increased ; the formal opening of the Home has yet to take place. Now that our Queen has civil nurses who bear her name, surely she will institute some order of merit, similar to the Royal Red Cross, but which can be attained by those who are not in the naval or military service. The many unknown and soon forgotten deeds of heroism within hospital wards deserve some recognition, that the force of example may encourage and help others.

AMERICAN DIRECTORIES.—Nursing Institutions are unknown in America ; the supply of private nurses is carried on by directories which charge both nurse and patient a small fee when an engagement is concluded, but the nurses take their own earnings. We have before given full particulars of the Directory at Philadelphia, and now a kind correspondent sends us an account of the Directory at Buffalo. There are now entered on the register of this Directory for Nurses forty-two trained nurses, and twenty-one untrained, except by the training gained from long experience. A movement is on foot among the younger graduates to raise the scale of prices from 15 dollars a week to 20 dollars and 25 dollars, and many of them have already signified their intention of not working for less than 20 dollars weekly. Miss Sara P. Sheldon is in charge of the Directory, and in the course of an interview she said :—"The nurses become impatient if they do not receive a call soon after registration, and many of them seem to think that I have cases hanging about the room like suits of armour to fit each individual if only I will take them down. One nurse wants a 'typhoid' case, another is unwilling to do anything but surgical work ; so, though there are nurses and cases, they will not always suit one another. Yes, we send many of our nurses to out-of-town cases. When a nurse returns from a case we expect her to notify the Directory immediately, that whatever after call she has may come through us, that we may have the benefit of the patient's fee. The nurses have nothing to pay after the initiation fee of 5 dollars, which is exacted from all. Physicians sometimes are careless and send a nurse from case to case without giving the Directory the benefit of this much needed fee."

A NURSE IN TROUBLE.—In last week's *Lancet* an account was given of the death of a patient in the Cork Fever Hospital from an overdose of paraldehyde. The deceased, Bridget O'Brien, aged twenty, was admitted suffering from typhoid fever, and but slight hopes were entertained of her recovery. As she was suffering from violent delirium and sleeplessness, a hypnotic was prescribed, and the temporary nurse, acting for the ordinary nurse during leave of absence, whose name was Horgan, was instructed, both verbally and in writing, how to administer the medicine. The patient getting worse, Horgan overlooked the directions she had received, and, instead of giving one teaspoonful, unfortunately administered the entire contents of the bottle—viz., from six to seven teaspoonfuls. In about five minutes O'Brien fell into an unconscious state, and, despite medical assistance, remained in that state for four hours, when she died. Horgan, it may be added, had done similar duty as temporary nurse in the most satisfactory manner during the past three years, and was a paid official of the hospital. She stated that, in the excitement caused by the delirium of the patient, she never looked at the label on the bottle. She has been arrested, bail being accepted, and the trial will take place shortly. Ireland is unlucky—the last case of misadventure to a patient of which we heard hailed from the Emerald Isle. Every nurse, during her period of training, should attain the unalterable habit of reading the label of every medicine bottle every time she uses it. It is an easily-learned custom, which sisters would do well to enforce by example and by precept ; the risk of misadventure is then greatly decreased.

### Sketches of Insanity.

By Francis H. Walmsley, M.D. (Senior Assistant Medical Officer of Leavesden Asylum).

### VIII.—MENTAL STUPOR.—HYPNOTISM.

One of the most distressing forms of insanity is the condition known as mental stupor; the patient is affected with a species of general torpor which causes his vital energies to sink below their normal pitch; he presents a more or less complete passivity, an apathy and profound indifference for all that comes in contact with him.

Mental stupor supervenes after a severe mental or moral shock, women are attacked more frequently than men, the majority of cases occur about the age of twenty, all mental power is suddenly arrested or entirely abolished, the mind becomes, more or less, a blank, the patient appears to be utterly incapable of making any effort, all energy and will-power seem to have vanished, external impressions excite no response, no effort can elicit either word or deed, the patient is perfectly mute to all questions, she appears to see nothing, to hear nothing, to feel nothing; if spoken to loudly she does not turn her eyes, if pinched or pricked she does not flinch, she has become a silent automaton—motionless, speechless; she is lost to all healthy feelings and human interests; if moved out into the room she stands like a statue, with blank stare, and gaze fixed right forward; set down at table and food placed in her mouth she eats, in like manner liquids are swallowed, all her bodily wants are attended to by others; if the arms be extended or raised above the head, they remain in this position for a length of time, or gradually drop down to the side, not unfrequently there is manifested cataleptic fixation of the limbs and body generally; certain patients suffer from an occasional access of terror and excitement, and behave as though they saw and heard imaginary objects about them, again relapsing into stupor; such patients are generally labouring under some frightful delusion which utterly sways their consciousness and will; in the majority, however, no sign of definite delusion appears—no violent impulse or speech, no outrageous word or deed. On recovering from their abnormal state such patients may have only the most dim and hazy remembrance of a vague feeling of strangeness, apathy, and powerlessness by which they were possessed.

The states of lethargy, catalepsy, somnambulism, and hypnotism are closely allied, if not identical with one another.

*Hypnotism.*—A consideration of the highly interesting and instructive experiments carried out by physiologists (Foster, Ferrier, Bastian, and others) will help us to a clearer understanding of the phenomena of hypnotism. If in a brainless frog a drop of acid be placed on its right flank, the right foot is almost invariably used to rub off the acid. In this there appears nothing more than a mere mechanical or automatic action; if, however, the right leg be cut off, or the right foot be otherwise hindered from rubbing off the acid, the left foot is, under the exceptional circumstances, used for the purpose. This at first sight looks like an intelligent choice. A choice it evidently is, but manifestly the frog cannot be conscious of exercising this choice; automatic life has replaced his conscious existence. Again, by the application of appropriate stimuli, a brainless frog can be induced to perform all the movements which an entire frog is capable of executing; it can be made to swim, to leap, and to crawl; when placed on its back it immediately regains its natural position; when placed on a board it does not fall from the board when the latter is tilted up, so as to displace the animal's centre of gravity; it crawls up the board until it gains a new position, in which its centre of gravity is restored to its proper place. Its movements are exactly those of an entire frog, except that they need an external stimulus to call them forth, they inevitably follow when the stimulus is applied. Again, if the frog's flanks be gently stroked it will croak, and the croaks follow so regularly and surely upon the strokes that the animal may almost be played upon like a musical instrument. If, in removing the brain of the frog, that portion which is concerned with the sense of sight be left undisturbed, we see that the movements of the animal are influenced by light; if it be urged to move in any particular direction, it will avoid in its progress objects casting a shadow. In some hypnotic subjects the excessive power of sight (as shown by Dr. Luys) reaches such an extraordinary pitch of acuteness that if we cover the eyelids with a layer of cotton wool, and then put a newspaper in front of the eyes, we are amazed to see that the person hypnotised can read it, no doubt through some tiny cracks imperceptible to us.

All that the patients do in the several states of somnolence or hypnotism is simply the result of automatic action—the mind is absent throughout. We saw that the brainless frog has become a machine, and nothing more. This machinery is put into action, and its movements originated and guided by a stimulus supplied from without, the stimulus passing along the sensory nerves of the skin, the eye, or the ear, as one or the other is played upon by the operator; this is precisely what takes place in the case of the hypnotised subject, who may be said to be in a condition analogous or similar to that of the brainless frog, so far, at any rate, as his consciousness is concerned.

He is in a trance-like state in which the ordinary functions of the mind are suspended—the reason, judgment, and will being in complete abeyance. In somnambulism, and to a less extent in ordinary dreams, we act our ideas out to the full, the usual restraining power being dormant; in hypnotic sleep the patient is open to the reception of ideas suggested by another person, while the mind is unsusceptible to the external situation generally, and is, to that extent, asleep. In ordinary sleep, the sleeper is, so to speak, wrapped up in himself. The hypnotic state differs from ordinary sleep in that there exists a relation between the sleeper and the operator; in this condition there is forgetfulness of the outer world, and a sleep is produced in which suggestions are readily acted on. In some cases sleep is partial in its area; portions of the brain that are usually involved in sleep remain exempt, so that the sleeper exhibits powers which sleep usually annuls—hence, we may have sleep-talking and sleep-walking. In sleep-talkers, dream thoughts are capable of evoking correlative acts of speech, and such persons will allow a listener to hold a sort of conversation with them, of which in the waking state they recollect nothing. In sleep-walking, again, we see that a person may get up, dress himself, go downstairs, put on his coat and hat, unlock the door, walk some distance, return, again undress, and retire to bed, and on the following morning may have no recollection whatever of what happened during the night. Certain faculties of his brain, unknown to himself, have been in a condition of exalted activity. The alliances here are intimate with hypnotism. In somnambulism the patient acts in obedience to suggestions from within; the stimulus is intrinsic. In hypnotism he acts in obedience to suggestions from without; the stimulus is extrinsic. In both states his consciousness is suspended.

*(To be continued.)*

### Owed

*(To a Charge Nurse).*

One more probationer,
  Weary and worn,
Gone to her cubicle,
  Dreading the morn.

Wake her remorselessly,
  Tell her she's slow,
Scold her, remind her
  She's only a Pro'.!

Has she intelligence?
  Tell her she's proud,
Bully her thoroughly.
  Soon she'll be cowed.

Does she risk an opinion?
  Squash her at once;
A junior probationer is
  Only a dunce.

Would she ask any questions?

  Snub well from the first,

And show it's no merit

  For knowledge to thirst.

Bid her run, fetch, and carry,

  Tell her not to be late;

Send her up with the jug

  And down for the plate.

Can she tell a good story?

  Then turn up your nose,

Charge Nurses don't listen

  To jokes from the Pro's.

Just one word of warning,

  And then you may go—

Revenge may be taken

  By—even a Pro'.!

### Why Hospital Authorities Object to the B. N. A.

Miss Lückes, in her evidence before the Lords' Committee on the 10th of July, said: "I am not in favour of what is called the British Nurses' Association for the Registration of Nurses. It is a terrible mistake. I think it is doing everything to retard the progress that nursing has been making." Asked why, Miss Lückes said: "It places good and bad nurses on a level. It is excellent for bad and inefficient nurses, and fatal to the good ones. Take the tests they might apply; it cannot be true that time is the test, or Miss Page would have been a splendid nurse. Or, if you think of provincial hospitals, three years in a small, quiet provincial hospital is not to be compared to the value of six months' experience at a London hospital; they would see so much more there. Then the theoretical examination, which is another test they might apply, is no guide whatever to the practical fitness of a woman for her work. It is my experience, and that of many other matrons, that those who come out best in their theoretical examinations are the least fitted either for good nursing or for managing a ward. You can no more make a nurse of a woman who has not a gift for nursing than you can make a musician of a person who has no ear for music, and no notion of the thing. Then I think that anything which places them all together on a register like that, when you have no distinct, definite, reliable basis, must make it more difficult even than it is at present to know whether you can obtain reliable women, or whether the qualifications are imaginary as it were. If I might, I should like to complete the account of the registration that we ourselves make. We copy the certificate into the probationer's register, we sum up her various qualifications. If she is appointed a staff nurse or private nurse she is so entered in the register, and a record is kept there; and, finally, at Miss Nightingale's suggestion, we have established a supplementary register, and when a nurse leaves she is given a paper which informs her that if she chooses to take the trouble to send me word what she is doing and how she gets on, her record of that will be entered for her benefit in this supplementary register. I am thus enabled to make inquiries as to what she has done in the interval, and to help her on further, or to give full information concerning her. Therefore, every nurse who once gets a hospital certificate can get every good from her own hospital, and the standard by which she is judged is very real all round."

### The Patient.

Who comes in black and needs a scrub,

Who says he never has a tub,

Who makes my arms ache as I rub?

The patient!

Who says, "I never slept a wink,"

Yet scarcely woke to take a drink,

And snored till breakfast-time, I think?

The patient!

Who pulls his bedclothes all awry,

To catch the passing matron's eye,

Whose locker, full of crumbs I spy?

The patient!

Who calls me just as I've sat down,

To say he wants his toast done brown,

Who thinks I never ought to frown?

The patient!

Who yet to me is very dear,

Whose better health my heart doth cheer,

Who, when he's ill, comes always here?

The patient!

### Everybody's Opinion.

[*Correspondence on all subjects is invited, but we cannot in any way be responsible for the opinions expressed by our correspondents. No communications can be entertained if the name and address of the correspondent is not given, or unless one side of the paper only be written on.*]

### ASYLUM NURSES.

"Only an Asylum Nurse," writes: A lot is being said of sick nurses and lunatics; let us hope the nurses in lunatic asylums will not be forgotten. The trials of asylum nurses are not as widely known as those of sick nurses, and they need sympathy and help equally as much, if not more. A good conscientious nurse must have a love of the work. She must be patient, self-denying, with plenty of tact, physical strength, and a good moderate education. Only those who have lived with the mentally afflicted can fully understand what is required of the nurse; the many different characters and ideas she has to contend with are very trying.

A mental nurse risks her life *and reason* in undertaking such a task. She must have a cheerful, flexible mind; she must always be on the alert for a change in the patient, often getting her full share of expletives and blows intended for an imaginary being. Experience teaches her good temper is a necessary, for which she gets little pleasure, small wages, and *few* thanks. A mental nurse also thwarts her own education, there being no possible chance of acquiring any help from the insane, the only time available for that being the hours off duty, generally two hours every day and half-a-day every week, with annual leave, when a change of scene and sound relieves as much as the rest. Fresh patients often enter in a weak bodily state; these recoveries must be a credit to the doctors and nurses as they labour under difficulties. I am one of the nurses, and I have often had it said to me, "Oh nurse, how can you do it, it would worry me to death," and yet we are not recognised as fellow helpers of human suffering.

Nurses are not coarse in themselves, as many consider them, although constant contact with the insane helps to corrupt much that is good. The more gentle and winning a nurse makes herself, the more influence she has over the patients.

I hope the time is short when mental nurses will receive their just due from the public so greatly benefited, and that those who will, may have the opportunity of learning all the grades of nursing, mental and physical.

### A DISCLAIMER.

Miss Augusta M. Powell, of the Laburnums, Salisbury, writes: I should be glad if you would state in The Hospital that I am not the "Nurse Powell" mentioned in connection with her temperature "before the Lords' Committee." May I add that though I have been in the nursing world several years, my happiest nursing time was my two years' probation at the London Hospital.

### LIFE AT JOHANNESBURG.

"Wyvern" writes:—I observe in last week's Hospital under "Scraps and Gleanings," a note referring to the mortality at Johannesburg. The figures given are correct, but the following extract from a letter from a lady residing at Johannesburg, will put another face on the state of affairs: Extract from letter dated July 3rd. "There is a ball at Doorfontein Club to-night for the benefit of the Nurses' Home; they have had no patients for some time, and are going out to nurse. The health of Johannesburg has never been so good. The nurses are in want of funds positively. Ladies sending supper dishes. We have made a nice little lot and sent down, &c., &c." On submitting this to a gentleman well acquainted with the position of affairs at Johannesburg, and whose statements can be absolutely relied upon, he submits the following comment:—"I assume it is a subscription ball, and that to conserve the receipts the ladies are providing the supper. The death rate last year amongst the European population was 40 per 1,000; the chief causes being typhoid fever, septic pneumonia, and virulent diarrhœa; the causa causans being the communal insanitariness attendant on flopping down some 40,000 people in a mining camp in the erstwhile wilds, also to the low vitality of many of the usual camp loafers and fast livers. The town itself stands on a plateau 6,000 above sea level, and its death rate soon should only be normal, *i. e.*, as soon as the new muncipal council gets a grip of the varied temples of cloacae and wields the trident of stercules in nineteenth century fashion."

### THE ATTACK ON THE LONDON HOSPITAL.

Miss Black writes: It is a pity that the discontented pros. who are still wailing in print do not first agree as to their statements. Miss Guthrie Smith writes that she was at the London at the same time as Miss Yatman, and she was put into a ward of 17 beds; the staff consisted of a probationer acting as staff nurse, a junior probationer, Miss Smith, and the sister of the ward, who overlooked all. Then Miss Smith goes on to repeat the old wails about the overwork, the bad food, &c., all of which has been distinctly and emphatically contradicted by men and women of long experience in hospitals and of good repute. (Miss Smith was a three months' probationer.) In the same journal in which this letter appears, Miss Yatman returns to the charge, and in an extraordinary table tries to prove that the allowance of nurses to patients at the London Hospital is about 1 to 20! This is most amusing, in face of the "corroboration" of Miss Smith, and the demonstrable fact that the nursing staff of the London Hospital is 218, and the number of beds occupied on an average 640. But what is not amusing is the way in which Miss Yatman arrives at her wonderful table, it is such a proof of the manner in which all these charges are trumped up. Miss Yatman ignores night nurses, sisters, &c.; she divides probationers in half (poor pros. !); she ignores the fact that the beds are not always all occupied, and after all, to be nurse over a ward which contains 13 *beds*, which are half of them empty, is not hard work. I will gladly undertake to nurse 20 *beds*, for I find they require very little attention. Miss Yatman is not wise; tables like this throw discredit on the evidence of anyone who could put them forward, and I hope they will cause Lord Sandhurst to smile. Miss Yatman should employ the vacation in banting and living in a dark cellar; then, perhaps, when the committee meets again, she would more appropriately look the overworked pro., broken down in health.

### REGISTRATION OF MIDWIVES.

"E. B." writes:—While there is so much being done now in the way of getting good nursing for the sick poor, I wish that many of our friends could realise how difficult it is to get the poor to avail themselves of the help which is generously offered to them. I am referring to the matter of providing trained midwives to attend poor women in their own houses either in rural districts or in towns where there are no lying-in-hospitals, We all know how often it happens that the poor woman dies from the treatment she sustains at the hands of those rough women so-called midwives, who go about attending the wives of the labouring class in their time of peril, and fill in their own time with field-work, carting and even coal carrying. A case is before me in this part of the country, I grieve to say it is only one of many, where a trained midwife is provided by ladies to go to several villages within a given radius, and the fee charged for her attendance is no larger than these other women readily obtain, and yet in spite of all kind persuasions, pointing out the benefits, etc., many of these poor country women refuse to avail themselves of the trained midwife's services. Although they confess that they would like to, "that Mrs. ——— takes snuff and is not very clean, still she has done for me a good few times and I don't like to give her up." These words were said to me quite recently by a most respectable-looking woman who also states that she nearly lost her life at her last confinement from hæmorrhage, the midwife, not knowing the danger, having neglected to send for the medical man at the proper time. I think, a fear of seeming ungrateful to an old friend, an inherent clinging to old customs, and a dislike to new faces are among their reasons for the course they persist in following. Only one thing can help us to help them in this matter even if need be, against their own wishes, until they know better; that is "The Midwives Registration Bill," which will compel all these women to become trained or to give up their untrained work, making them answerable to law if they do as they are doing now. Oh! that everyone who has the welfare of the poor at heart would do their utmost to get the Bill passed.

### Notes and Queries.

To Correspondents.—1. Questions may be written on post-cards. 2. Advertisements in disguise are inadmissible. 3. In answering a query please quote the number. 4. If a private answer is desired, a stamped addressed envelope must be forwarded.

Notice.—We must really ask our correspondents to be more particular in enclosing name and address. Constantly we receive queries or letters with no name attached.

### Queries.

(33) *St. Katherine's Hospital.*—Is St. Katherine's Hospital a home for trained nurses when old, or temporary for the Queen's Nurses when disengaged?

### Answers.

*Lowestoft.*—We have lately published letters in favour of Canada, which state the autumn is the best time to go.

*Eva Graham.*—See the list of medical staff to the Hereford and Hertford hospitals in the *Hospital Annual.*

### Presentations.

There was a very pretty scene at Glasgow Royal Infirmary on August 18th, when Miss Wood, the Matron, was the recipient of a silver cake-basket and a gong, with the following inscription: "Presented to Miss Wood, by the members of her class, on her birthday."

On July 31st a presentation was made by the nurses of the Royal Portsmouth Hospital to Sister A. R. M. Sharp, for two and a-half years head nurse at that hospital. She was presented with a handsome diamond ring as a sign of the deep regret felt by the nurses at her departure. Sister Sharp received splendid testimonials from the medical staff. The Royal Portsmouth Hospital will feel the loss of Sister Sharp for some time, as she was highly valued by the medical staff, beloved by the nurses, and respected by the patients.

### Vacancies.

The following vacancies are announced:

**Matrons.**

Weymouth College.
Wallasey Fever Hospital; £35.

**Sister.**

Borough Fever Hospital, Leeds; £32.

**Nurses.**

Blackburn Infirmary (Night, Charge); £27.

Bromley and Beckenham Hospital; £25.

Bournemouth Institution; £25.

Blackheath and Richmond Institutions.

Chelsea Infirmary; £18.

Cambridge Home.

Cheltenham Institution.

Fleming Memorial Hospital for Children, Newcastle-on-Tyne.

Greenwich Union; £20.

Haywood Hospital, Burslem; £23.

Hanover Institute; £25.

Kingston Home, Hull.

Kingston-upon-Hull Incorporation for the Poor; £30.

Levick Institution, Paris.

London and Brighton Association, Brighton.

Lowestoft Association; £26.

Mercer's Hospital, Dublin; £20.

Merthyr Hospital; £20.

Monkwearmouth and Southwick Hospital; £25.

N.R. Infirmary, Middlesboro'; £25.

Nurses' Home, Hull.

Nursing Institution, Wimpole Street, W.

Nursing Institution, Ventnor, I.W.; £25.

Ophthalmic School, Hanwell; £26.

Ditto (Assistant); £18.

Surrey County Asylum, Woking; £16 and £18.

St. George's Home, Sheffield.

St. Saviour's Infirmary, Dulwich; £20.

St. Helena Home, N.W.

Tunbridge Wells Hospital (Night); £25.

Torquay Institution.

Victoria Home, Bournemouth; £25.

Worthing Infirmary; £24.

Workhouse Infirmary Association.

**District Nurse.**

Nurses' Home, Leicester; £52.

**Probationers.**

Boston Hospital, Lincolnshire.

Clayton Hospital, Wakefield.

H.M. Hospital, Stepney.

Hospital for Chronic Children, N.

St. Helena Home, N.W.

Workhouse Infirmary Association.

### Amusements and Relaxation.

### SECOND QUARTERLY WORD COMPETITION.

Commenced July 5th, 1890, ends Sept. 27th, 1890.

Three prizes of 15s., 10s., 5s., will be given for the largest number of words derived from the words set for dissection.

Proper names, abbreviations, foreign words, words of less than four letters, and repetitions are barred; plurals, and past and present participles of verbs, are allowed. Nuttall's *Standard* dictionary only to be used.

N.B.—Word dissections must be sent in WEEKLY not later than the first post on Thursday to the *Prize Editor*, 140, Strand, W.C., arranged alphabetically, with correct total affixed.

The word for dissection for this, the NINTH week of the quarter, being

"GROUSE."

| Names. | Aug. 21st. | Totals. |
|---|---|---|
| Dulcamara | 80 | 480 |
| Tinie | 83 | 502 |
| Henri | — | 395 |
| Agamemnon | 84 | 510 |
| Patience | 83 | 502 |
| Brisbane | — | 332 |
| Nurse Hilda | 83 | 482 |
| Lightowlers | 74 | 495 |
| Jenny Wren | 80 | 462 |
| Rhoda | — | 242 |
| Annabel | — | 17 |
| Spare Moments | — | 19 |
| Qu'appelle | 79 | 467 |
| Nurse | — | 29 |
| A. B. C. | — | 251 |
| Brickbat | — | 77 |

**Notice to Correspondents.**

N.B.—Each paper must be signed by the author with his or her real name and address. A *nom de plume* may be added if the writer does not desire to be referred to by us by his real name. In the case of all prize-winners, however, the real name and address will be published.

Competitors can enter for *all* quarterly competitions, but no competitor may take more than *one first prize* during the year.

